# An Atypical Variant of Apple Peel Atresia: Reporting a Rare Case

**DOI:** 10.7759/cureus.6047

**Published:** 2019-11-01

**Authors:** Taha Bin Arif, Jawad Ahmed, Farheen Malik, Sharmeen Nasir, Aiman Ali

**Affiliations:** 1 Internal Medicine, Dow University of Health Sciences, Karachi, PAK; 2 Paediatrics, Dow University of Health Sciences, Karachi, PAK

**Keywords:** apple peel atresia, jejunoileal atresia, congenital malformation, gastrografin study

## Abstract

Apple peel intestinal atresia is a rare congenital malformation. It consists of a proximal jejunum ending in a blind pouch and distal small bowel wrapped around its vascular supply in a spiral fashion. A combination of type IIIb jejunoileal atresia (apple peel atresia) and type IV (multiple intestinal atresias) is a rare entity. The diagnosis and management of such complicated cases is a challenge, especially in resource-limited settings. We report a case of a four-day-old female who presented to the neonatal intensive care unit with complaints of vomiting, yellow discoloration of the skin, and failure to pass meconium since birth. The baby was born preterm (34 weeks) via spontaneous vaginal delivery. The physical examination concluded a jaundiced and dehydrated child with a soft, non-tender abdomen and absent gut sounds. X-ray abdomen showed two air-fluid areas in the left hypochondrium. The upper gastrointestinal gastrografin study revealed that contrast opacified the third part of the duodenum and no contrast was observed beyond it. On exploratory laparotomy, proximal jejunal atresia with four distal atresias in apple peel fashion and a viable 20 cm of small bowel was observed. The apple peel segments were supplied by mesenteric vessels. Unfortunately, our patient expired despite all supportive measures. The case highlights the significance of the prenatal and early postnatal diagnosis of such a complex combination of intestinal atresias for adequate and timely management.

## Introduction

Jejunoileal atresia (JIA) is a congenital defect in the small bowel. Along with atretic segments, it is characterized by the partial or complete absence of membranes connecting the small intestines to the mesentery producing a significant loss of bowel length. Apple peel atresia also known as Christmas tree atresia is a rare variant of JIA with an incidence of 0.7%-0.8% in 10,000 live births [1]. It consists of proximal jejunal atresia and a short segment of ileum spiraling around its vascular supply. Although there have been recent advances in the outcome, it is associated with higher morbidity and mortality rates [2]. Apple peel atresia is usually reported as an isolated malformation, however, it has also been related to malrotation, situs inversus, and polysplenia [3].

 A thorough review of the literature reveals that only a few cases of apple peel atresia have been reported in Pakistan [4-5]. However, so far, multiple intestinal atresias coexisting with apple peel deformity have never been reported in South Asia to the best of our knowledge. Therefore, the rarity, diagnostic quandary, and poor prognostic outcomes of such special variants of JIA make this an interesting case to report. Here, we report a case of a four-day-old female from Pakistan who presented to the neonatal intensive care unit (NICU) with complaints of vomiting, yellow discoloration of the skin, and no passage of meconium since birth.

## Case presentation

A four-day-old female neonate, unvaccinated, weighing 1.7 kg, presented to the newborn intensive care unit (NICU) at Civil Hospital Karachi (CHK) with complaints of vomiting, failure to pass stool after birth, and yellow discoloration of the skin since the first day of life. The patient had multiple episodes of non-projectile, non-bilious vomiting with no fever. Jaundice was progressively increasing for the last four days and no stool or meconium was passed per rectum. She was on direct breastfeed. The patient’s antenatal and natal histories were insignificant. The baby was born preterm (at 34 weeks of gestation) via a simple, spontaneous, and uncomplicated vaginal delivery at a local hospital with an immediate cry at birth. The mother was well throughout the pregnancy and did not report the use of any medications. Two ultrasounds were done in the first and second trimester, which did not show any anomaly. The patient was the third progeny of a consanguineous marriage. Parents and all other siblings were alive and healthy.

The general physical examination concluded an icteric looking, lethargic, tachycardic, and dehydrated neonate with jaundice up to mid-thighs. Anthropometric measurements showed a low birth weight (1.7 kg), birth length (41 cm), and fronto-occipital circumference (29 cm). A soft, non-tender abdomen with absent gut sounds was found on the abdominal examination. The rectum was empty on digital rectal examination. Neonatal reflexes were poor. No significant findings were noticed in the examination of other systems.

Small intestinal atresia (duodenal or jejunal atresia) and malrotation were kept as differentials in the diagnosis considering non-bilious vomiting and no passage of stool with an empty rectum. Laboratory investigations were carried out to reach a final diagnosis. Complete blood count (CBC) revealed a normal hemoglobin (Hb) level of 15.4 gm/dL, a raised total leukocyte count (TLC) of 12 x 10^9^/L, and a normal platelet count (PLT) of 353 x10^9^/L. Serum electrolytes were done, which showed hypokalemia of 2.4 mEq. Renal function was impaired with an elevated blood urea nitrogen (BUN) of 47 mg/dL and creatinine of 1.7 mg/dL. C-reactive protein (CRP) was also well above normal limits (32 mg/dL). Liver function tests (LFTs) showed elevated direct bilirubin and total bilirubin of 0.91 mg/dL and 12.4 mg/dL, respectively.

**Figure 1:**
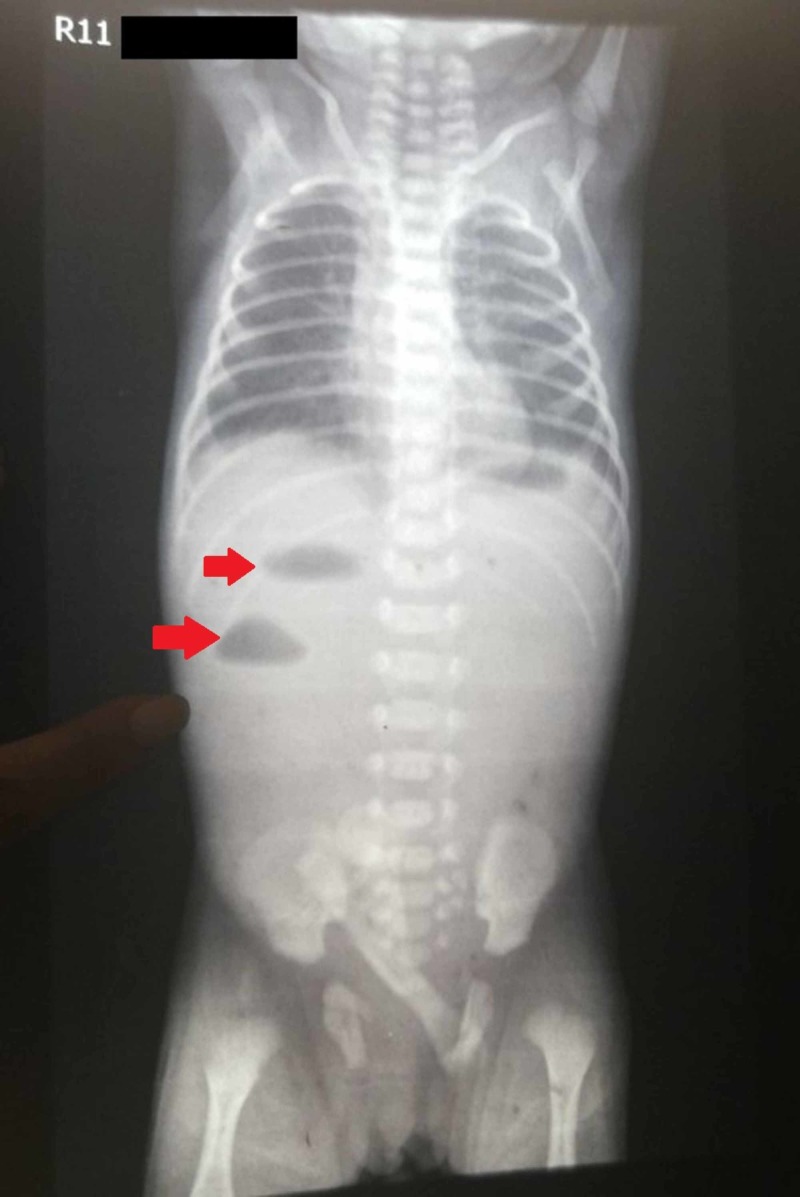
X-ray abdomen showing two air-fluid levels in the left hypochondrium

**Figure 2:**
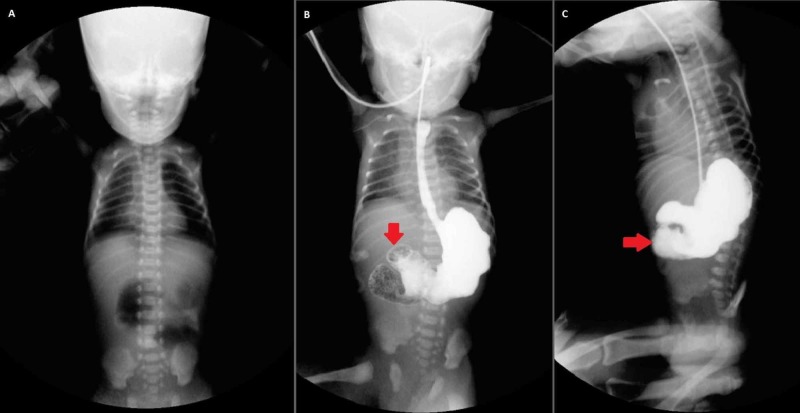
Upper gastrointestinal gastrografin study showing proximal jejunal atresia. The contrast opacified the stomach up to the third part of the duodenum beyond which no contrast is observed Figure 2A: Before contrast. Figure 2B and 2C: After contrast

X-ray abdomen showed two air-fluid levels in the left hypochondrium while the rest of the abdomen was gasless (Figure 1). The upper gastrointestinal gastrografin study revealed that the contrast opacified the stomach and third part of the duodenum beyond which no contrast was seen (Figure 2). A pediatric surgery consult was sought. Jejunal atresia was suspected and laparotomy was planned. The patient was initially kept nil per oral (NPO) and intravenous (IV) antibiotics, namely, cefotaxime, amikacin, and metronidazole were started. Nasogastric (NG) tube decompression was carried out. The patient was rehydrated with ringer lactate (RL) and dextrose sugar with the addition of potassium chloride (KCL). Neonatal gastrointestinal losses were replaced with an equal amount of normal saline (NS).

**Figure 3:**
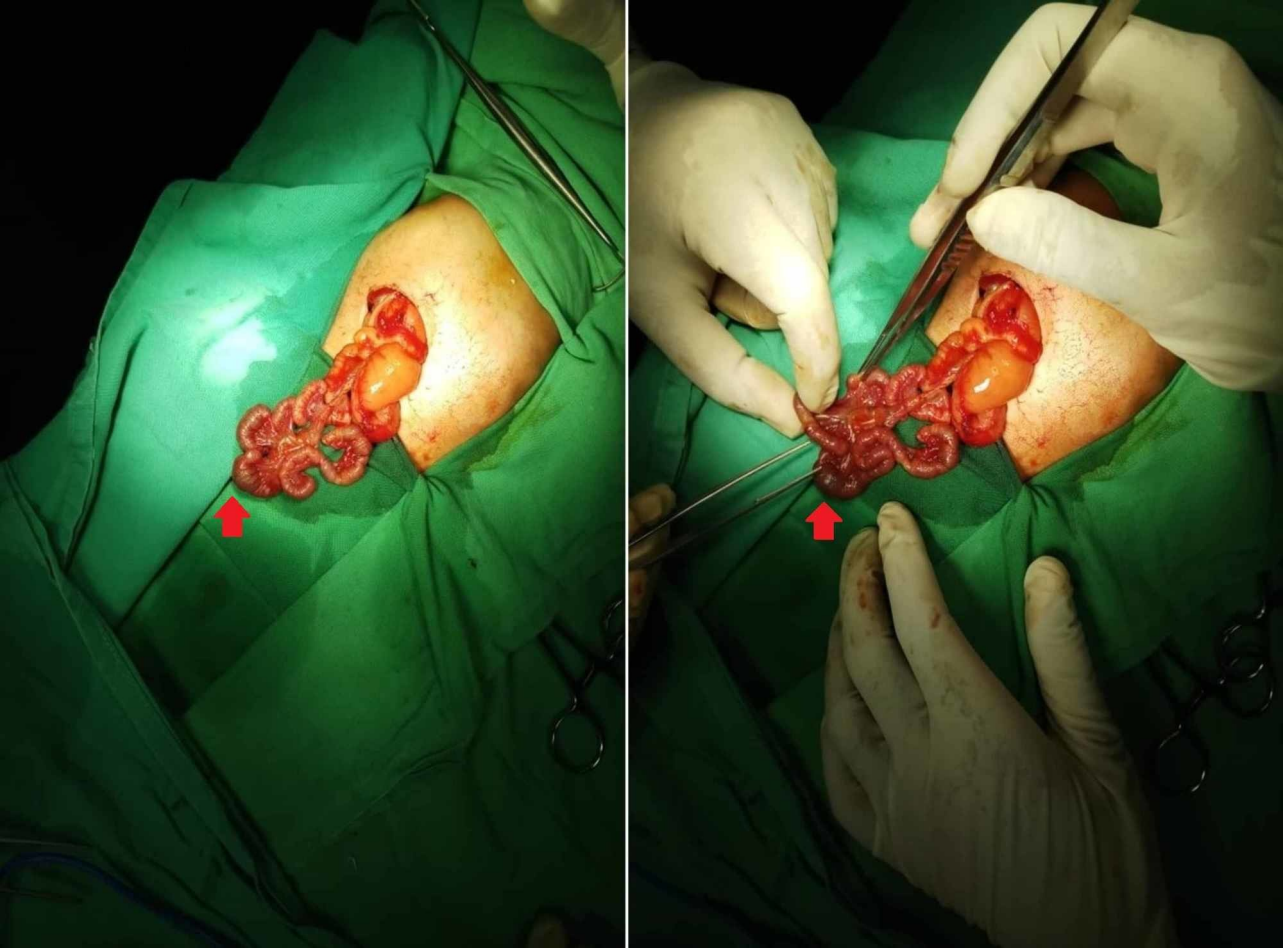
Exploratory laparotomy showing a dilated duodenum and proximal jejunal atresia with multiple segments within type IIIb (apple peel) atresia

**Figure 4:**
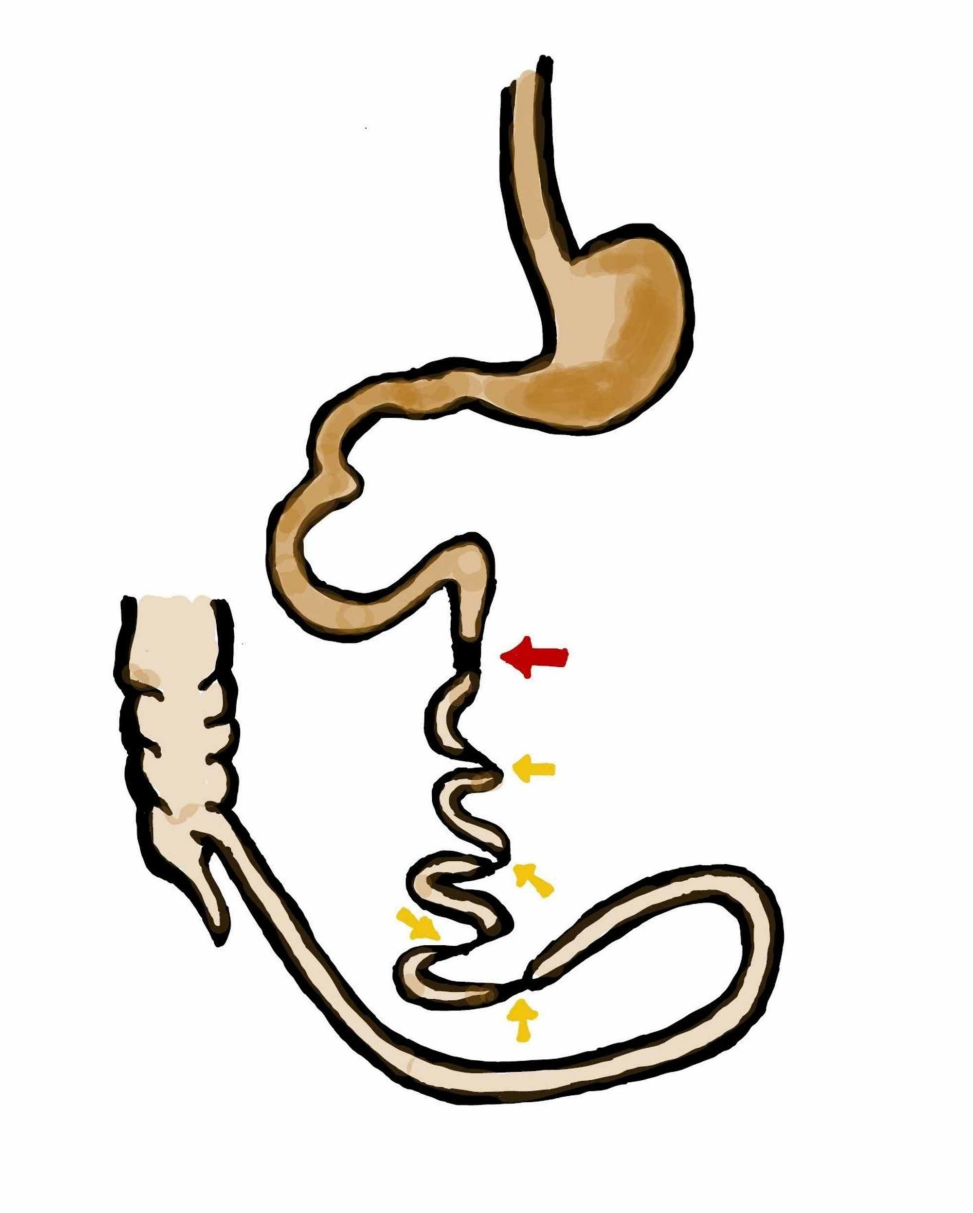
Diagrammatic representation of pathology showing proximal jejunal atresia (red arrow) with distal four atresias within type IIIb atresia (yellow arrows)

Exploratory laparotomy was performed after stabilizing the patient, which concluded type IIIb intestinal atresia with a markedly dilated distal duodenum and proximal jejunal atresia. The remaining small bowel was subsequently untwisted and the patient was noted to have four distal atresias in an apple peel fashion, with a viable 20 cm portion of the ileum. The apple peel segments were supplied by mesenteric vessels (Figure 3). The four distal atresias appeared as separate segments, as depicted in Figure 4. Due to the complexity of this case and the ominous prognosis of the condition, after a discussion with the parents, a decision was made to refrain from any further corrective surgery and the abdomen was closed. Supportive management and end-of-life care were continued for the patient. She expired on the seventeenth-day post-surgery.

## Discussion

A neonatal intestinal obstruction is commonly caused by intestinal atresia. Depending upon the location and number of atresias, the management and prognosis of the patient may vary. Louw and Barnard proposed a theory to explain the cause of intestinal atresia based on an experiment on dogs. They suggested that a mesenteric vascular accident in the later stages of intrauterine life may cause intestinal (jejunoileal and colonic) atresias [6]. Studies have found that JIA may occur due to intussusception, intestinal volvulus, strangulation in gastroschisis, or focal perforation secondary to localized inflammation [7-10].

Grosfeld et al. categorized JIA further into four types [10]. Type I is a mucosal defect and type II has atretic bowel ends connected by a fibrous cord. Type III has two variants: type IIIa has a V-shaped defect in the atretic segment and type IIIb is apple peel atresia where a single blood vessel nourishes proximal jejunal atresia and distal bowel. Type IV has multiple atresias giving a ‘string of sausages’ effect [10]. Type IIIb is the least common variant and is seldom encountered [11]. Our patient had an irregular type IIIb variant of JIA but with multiple (four) intestinal atresias arranged in an apple peel pattern. This pattern of atresia in the apple peel variant has not been reported previously.

All intestinal atresias usually present early in life or at birth with symptoms of bowel obstruction. They manifest as distension of the abdomen, failure to pass meconium, visible peristalsis, bilious vomiting, and respiratory distress (due to abdominal distension). Our patient presented with similar complaints of vomiting, failure to pass meconium, and yellowish discoloration of the skin. However, a soft abdomen with no abdominal distension was noted in our case and gut sounds were also absent. Various congenital anomalies are found in JIA patients, including malrotation, Meckel diverticulum, biliary atresia, cystic fibrosis, along with urogenital, cardiac, and neurological anomalies [11]. No such associated anomalies were identified in our case.

Almost one-third of JIA and duodenal atresia (DA) can be identified during the prenatal period with ultrasonography (US) [12-13]. Repeated US scans can be used to assess the progress and development of atresias in the fetal life period [13]. Cases of intestinal atresias that are diagnosed prenatally have more pronounced intestinal distention as compared to those diagnosed in the postnatal period [12]. This highlights the importance of prenatal scans as they can aid in the timely identification, management, and correction of defects, thus avoiding and saving the patient from developing life-threatening complications. Our patient had two normal prenatal US scans before the mid of the second trimester and no abnormality was detected at that time.

Different modalities, such as X-ray, US scan, and upper gastrointestinal gastrografin study, can be used for establishing a postnatal diagnosis of intestinal atresias including JIA [13-14]. The X-ray may show dilated bowel due to the presence of air. The US scan can provide valuable information about gut movements in addition to the presence of atresias. Similarly, a gastrografin study is used to confirm the diagnosis in cases where the US alone is not sufficient. Due to the presence of associated cardiac, urogenital, and abdominal anomalies, respective investigations, such as US and echocardiography, should be performed. In our case, the US was not performed. The X-ray showed two air-filled areas in the left hypochondrium and no contrast was seen beyond the third part of the duodenum in the gastrografin study. Other initial investigations include CBC, CRP, LFT, and renal function tests, which were also carried out in our case.

Based on the clinical signs and symptoms, investigations were done for DA, jejunal atresia, and malrotation. DA has a similar clinical presentation as JIA but the X-ray has a ‘double-bubble sign’; this was not seen in our patient’s X-ray. No malrotation was found on laparotomy.

After establishing the diagnosis, initial management includes placing a nasogastric (NG) tube and starting intravenous (IV) fluids and antibiotics [14]. Surgery is the preferred mode of treatment for JIA. The goal of treatment is to preserve as much bowel as possible. The operative procedure involves resection of dilated end of bowel and anastomosis with the distal end [2,11,13-15]. In some cases, resection of the bowel segment containing atresias may be done. Multiple anastomoses can be created to prevent a profound shortening of the bowel [14]. Corrective surgeries in JIA patients are followed by various postoperative (PO) complications, namely, sepsis, short bowel syndrome, necrotizing bowel, stenosis, and even mortality. PO mortality, as high as 11%, has been reported in the literature [11]. The prognosis of patients who survive PO morbidity caused by malnutrition can have normal growth and bowel functions [16].

After initial management and fluid resuscitation, an exploratory laparotomy was performed in our patient. It was seen that the proximal jejunum was atretic and four atresias were present in apple peel fashion distally. Only 20 cm of the remaining small bowel (ileum) was viable. Unfortunately, due to a lack of resources and adequate facilities for PO management, along with the complexity of the patient's condition and grave prognosis, no surgical correction was done (with parental consent).

## Conclusions

Our case reports a rare, complicated variant of apple peel atresia. Apple peel malformation can present in a complex form with various malformations and atresias of the bowel. Despite high mortality rates, it can be treated and managed with appropriate surgical procedures and excellent postoperative care. Our case highlights the importance of prenatal and early postnatal diagnosis of intestinal atresias so adequate measures can be taken to correct it as soon as possible. Appropriate parenteral nutrition also plays a vital role in determining the prognosis of patients. Limitations in terms of resources and facilities are an important negative factor in determining the prognosis of such complicated cases.

## References

[1] Best KE, Tennant PW, Addor MC (2012). Epidemiology of small intestinal atresia in Europe: a register-based study. Arch Dis Child Fetal Neonatal Ed.

[2] Lee SH, Cho YH, Kim HY, Park JH, Byun SY (2012). Clinical experience of complex jejunal atresia. Pediatr Surg Int.

[3] Chinya A, Naranje K, Mandelia A (2019). Situs inversus abdominalis, polysplenia, complex jejunal atresia and malrotation in a neonate: a rare association. Int J Surg Case Rep.

[4] Kamal A, Khan K, ur Rahman I, Khan A (2010). Small gut atresia in neonates. J Ayub Med Coll Abbottabad.

[5] Imran M, Rehman HU, Rehman IU, Waheed T, Khan I (2011). Outcome of Bishop Koop procedure in neonatal jejunoileal atresias: a retrospective analysis. KUST Med J.

[6] Louw JH, Barnard CN (1955). Congenital intestinal atresia observations on its origin. Lancet.

[7] Santulli TV, Blanc WA (1961). Congenital atresia of the intestine: pathogenesis and treatment. Ann Surg.

[8] Grosfeld JL, Clatworthy HW (1970). The nature of ileal atresia due to intrauterine intussusception. Arch Surg.

[9] Gornall P (1989). Management of intestinal atresia complicating gastroschisis. J Pediatr Surg.

[10] Grosfeld JL, Ballantine TV, Shoemaker R (1979). Operative management of intestinal atresia and stenosis based on pathologic findings. J Pediatr Surg.

[11] Stollman TH, de Blaauw I, Wijnen MH, van der Staak FH, Rieua PN, Draaismab JM, Wijnena RM (2009). Decreased mortality but increased morbidity in neonates with jejunoileal atresia; a study of 114 cases over a 34-year period. J Pediatr Surg.

[12] Basu R, Burge DM (2004). The effect of antenatal diagnosis on the management of small bowel atresia. Pediatr Surg Int.

[13] Rich BS, Bott M, Spigland N (2013). Multiple intestinal atresia with apple peel syndrome successfully treated with primary repair. J Pediatr Surg Case Rep.

[14] Gupta S, Gupta R, Ghosh S, Gupta AK, Shukla A, Chaturvedi V, Mathur P (2016). Intestinal atresia: experience at a busy center of North-West India. J Neonatal Surg.

[15] Balineni P, Kamal S, Manickam P, Shivaji K (2019). Apple peel atresia: a case report. Int Surg J.

[16] Festen S, Brevoord JC, Goldhoorn GA (2002). Excellent long-term outcome for survivors of apple peel atresia. J Pediatr Surg.

